# Cu-Ion Hybrid Porous Carbon with Nanoarchitectonics Derived from Heavy-Metal-Contaminated Biomass as Ultrahigh-Performance Supercapacitor

**DOI:** 10.3390/ijms26020569

**Published:** 2025-01-10

**Authors:** Jieni Wang, Xiaobo Han, Shuqin Zhang, Haodong Hou, Chenlin Wei, Chenxiao Liu, Leichang Cao, Jinglai Zhang, Li Wang, Shicheng Zhang

**Affiliations:** 1 Henan Key Laboratory of Protection and Safety Energy Storage for Light Metal Materials, College of Chemistry and Molecular Sciences, Henan University, Kaifeng 475004, China; jieniwang@henu.edu.cn (J.W.); 2138030016@henu.edu.cn (X.H.); zhangshuqin@henu.edu.cn (S.Z.); houhd@henu.edu.cn (H.H.); chenlinwei311@gmail.com (C.W.); lcxdxyyx@henu.edu.cn (C.L.); chemwangl@henu.edu.cn (L.W.); 2 Miami College, Henan University, Kaifeng 475004, China; 3 Department of Environmental Science and Engineering, Fudan University, Shanghai 200433, China; zhangsc@fudan.edu.cn

**Keywords:** heavy-metal-contaminated biomass, nanoporous carbon, copper-ion hybrid supercapacitor, ultrahigh-energy storage properties

## Abstract

It is challenging to handle heavy-metal-rich plants that grow in contaminated soil. The role of heavy metals in biomass on the physicochemical structure and electrochemical properties of their derived carbon has not been considered in previous research. In this study, Cu-ion hybrid nanoporous carbon (CHNC) is prepared from Cu content-contaminated biomass through subcritical hydrocharization (HTC) coupling pyrolytic activation processes. The CHNCs are used as advanced electrode material for energy storage applications, exhibiting an impressively ultrahigh capacitance of 562 F g^−1^ at a current density of 1 A g^−1^ (CHNC-700-4-25), excellent energy density of 26.15 W h kg^−1^, and only 7.59% capacitance loss after enduring 10,000 cycles at a current density of 10 A g^−1^, making CHNCs rank in the forefront of previously known carbon-based supercapacitor materials. These comprehensive characterizations demonstrate that copper ions introduce new electrochemically active sites and enhance the conductivity and charge transport performance of the electrode material, elevating the specific capacitance of CHNC from 463 to 562 F g^−1^. These findings offer valuable insights into the effective energy storage application of heavy-metal-contaminated biomass wastes.

## 1. Introduction

In the last decade, hyperaccumulators have been widely used in heavy-metal-polluted areas to be an effective method to remediate heavy metal pollution [1,2]. Therefore, the treatment and recycling of a large amount of generated heavy-metal-contaminated biomass is a significant environmental issue, which will avoid the waste of biomass resources and the secondary pollution of heavy metals into the environment [3,4]. For example, if casually discarded, the heavy-metal-contaminated biomass may undergo decomposition, resulting in the re-release of the heavy metal from the decaying biomass into the ecosystem, potentially inducing secondary pollution [5]. However, although many studies have focused on the screening and identification of hyperaccumulators, application and optimization of phytoremediation technologies, and ecosystem monitoring and risk assessment, there is still restricted information on the harmless disposal and recycling strategies for heavy-metal-contaminated biomass [6,7,8]. There is an urgent need for effective and environmentally friendly strategies to handle these plants’ biomass properly.

Subcritical hydrothermal carbonization (HTC) is a promising method for reducing and recycling biomass into biochar and various high-valued aqueous products in this process [3,9,10]. These products can be widely used in energy, materials, and other fields to realize the utilization of biomass resources. For instance, in comparison to raw biomass, biochar significantly reduces mass, facilitating storage, and can be further used as raw carbon material for the synthesis of other functional carbon materials [11]. More importantly, compared with other traditional technologies such as incineration and pyrolysis, HTC can effectively control the release of pollutants and fully recover high-value chemicals, heavy metals, and hydrochar [12,13,14]. In the process of hydrothermal treatment, the chemical speciation of heavy metals in biomass may change, transforming from bioavailable forms into stable forms, thereby mitigating the environmental hazards posed by heavy metals [15,16,17]. However, the profound influence of heavy metals on hydrothermal products was significantly overlooked, posing a severe challenge to the effective regulation and enhanced recycling of HTC products.

Recently, the excessive consumption of fossil fuels, including coal, oil, and natural gas, has triggered global environmental problems, encompassing energy crises and global warming, which have stimulated the exploration of new alternative energy sources [18]. Supercapacitors (SCs) with fast charging speeds, long cycle life, high energy and power densities, and green environmental protection provide a promising method for the rapid storage of renewable energy and show a wide range of applications in new energy power generation systems, consumer electronics, automotive transportation, and other fields [19,20]. Notably, hydrochar, as one of the HTC products of biomass, has demonstrated application potential in several fields, such as solid fuels [21] and porous carbon precursors used in carbon dioxide capture [22], water purification [23], and electrochemical energy storage [24]. The applicability of hydrochar heavily relies on its distinct physical and chemical characteristics. Simultaneously, the inclusion of heavy metals can exert a notable influence on these properties [25], potentially impeding their subsequent recycling. Nevertheless, studies on the impact of heavy metals on the physical and chemical attributes of hydrochar-derived porous carbon are quite restricted. In addition, due to nanoporous carbon materials derived from hydrochar generally having high specific surface areas, developed porous structures, and excellent electrical conductivity, they are frequently employed as electrode materials for supercapacitors. However, there is no relevant research about the influence of heavy metals present in hydrochar on the electrochemical performance of the porous carbon derived from it.

To fill this knowledge gap, we presented a refined approach involving hydrocharization of Cu-contaminated pine sawdust biomass, followed by pyrolytic activation, to prepare Copper-ion hybrid nanoporous carbon (CHNC) with different concentrations of heavy metals. To gain a deeper understanding of the effects of heavy metals, the morphological characteristics, surface functional groups, and pore size distribution of the as-synthesized hydrochar and porous carbon materials were investigated. Furthermore, the electrochemical performance of the CHNC was also explored. In addition to providing a new pathway for environmentally benign and large-scale preparation of high-performance electrochemical energy storage materials from heavy-metal-contaminated biomass, this study also expands the understanding of the role of heavy metals in the electrochemical behavior of carbon material.

## 2. Results and Discussion

### 2.1. Migration and Distribution of Heavy Metals and Their Effects on Hydrochar and Porous Carbon’s Elemental Compositions

Table 1 shows the Cu content and distribution in various carbon samples. As the content of copper (Cu) in raw pine sawdust continuously increased, the concentration of Cu pre-carbonized hydrochar gradually ascended. The Cu content in PSPH-25 and PSPH-50 is notably higher than that in PSPH-0, suggesting that the added Cu was enriched in pine sawdust pre-carbonized hydrochar, which is in agreement with the previously reported works on the hydrothermal liquefaction conversion of heavy-metal-polluted biomass under much higher temperatures (280–340 °C) [26,27]. The Cu distributions in CHNC-600-4-25, CHNC-700-4-25, and CHNC-800-4-25 were 7.51%, 13.64%, and 18.59%, respectively, indicating that an increase in temperature has a positive effect on the fixation of Cu in hydrochar and CHNCs. The Cu distributions of CHNC-700-2-25 and CHNC-700-4-25 were 14.53% and 13.64%, respectively, suggesting that an increase in the proportion of activator reduces the degree of stabilization of Cu in porous carbon. This is likely mainly because the increase in the mass of the activator made the state of CHNC looser, thereby leading to a change in the combination of Cu and porous carbon. Additionally, during the activation process, hydroxide ions in the activator could combine with Cu to form copper hydroxide precipitates. During the washing process, Cu would dissolve in acid and be partially removed, resulting in a significant reduction in the Cu content in porous carbon. Moreover, a higher temperature and a lower activator content are beneficial for the fixation of Cu in CHNCs.

**Figure 1:**
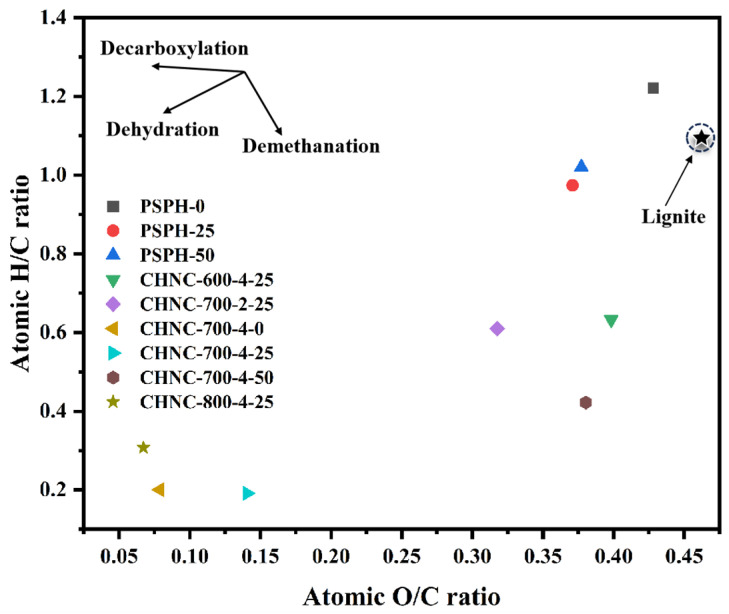
Van Kraveren diagram of hydrochars and porous carbons.

Table 2 shows the elemental compositions and yields of hydrochars and CHNCs obtained from heavy-metal-rich pine waste. As a typical lignocellulosic biomass, CHNC material had about 80wt% of C, which is much higher than other types of biomass (e.g., algae, manure, and food waste) [28], indicating a conducive formation of different morphological structures of the carbon skeleton, thus possibly affecting the electrochemical performance of the carbon material [29,30]. Figure 1 presents the Van Kraveren diagram of hydrochars and porous carbons, which includes the comparison of the various samples with lignite. After the hydrothermal reaction, in comparison with the hydrochar without a Cu addition, the percentage contents of nitrogen (N), hydrogen (H), and oxygen (O) in the hydrochar with Cu added were relatively decreased, while the percentage content of carbon (C) was relatively increased. The ratios of H/C and O/C exhibited a downward trend. This suggests that the addition of Cu may enhance the decomposition of oxygen-containing functional groups. This is mainly because Cu ions promoted the dehydration and decarboxylation reactions that were about to occur in the sample during the pre-carbonization process, thereby elevating the loss of H and O. The elemental properties of hydrochars are close to those of lignite. After the activation of hydrochar, as the temperature rises, the O/C ratio of porous carbon showed a decreasing tendency, while the H/C ratio showed a fluctuating trend. This is mainly because high temperatures could promote the pyrolysis of hydrochar and the aromatization of porous carbon, leading to the strong decomposition of oxygen-containing functional groups and a reduction in C-H bonds. As the Cu content increased from 0 to 50 mg/g, the percentage content of C in porous carbon was relatively reduced, the percentage contents of N, H, and O were relatively increased, and the O/C ratio showed an increasing trend, indicating that the addition of Cu enhanced the demethanation reaction in the porous carbon. Under different activator ratios, compared with CHNC-700-2-25, the percentage content of C in CHNC-700-4-25 relatively increased, the percentage contents of N, H, and O relatively decreased, and the O/C ratios significantly decreased, indicating that a higher activator ratio could assist the carbonization and aromatization of the carbon. Therefore, different temperatures, activator ratios, and Cu contents may alter the types and quantities of functional groups on the surface of porous carbon and the aromatization degree, thereby changing the electrochemical activity of CHNCs.

### 2.2. Other Physical and Chemical Characterizations of CHNC Materials

**Figure 2:**
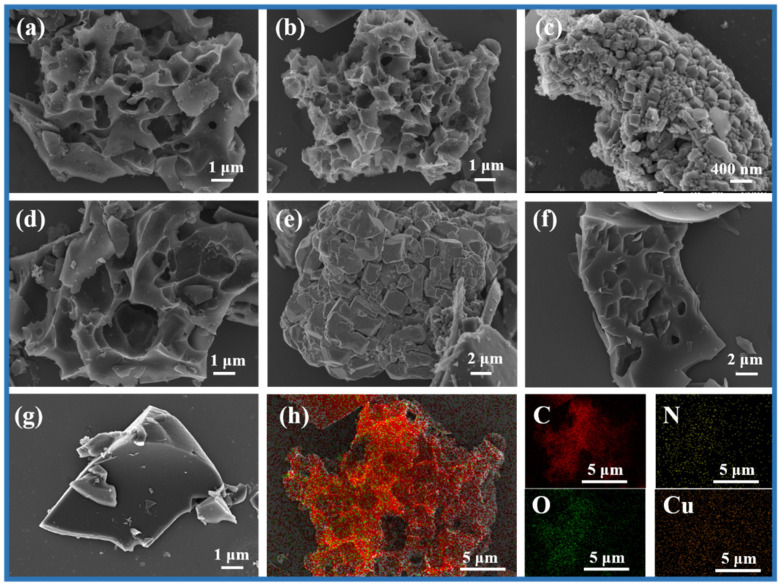
SEM images of (**a**) CHNC-700-4-0, (**b**) CHNC-700-4-25, (**c**) CHNC-700-4-50, (**d**) CHNC-600-4-25, (**e**) CHNC-800-4-25, (**f**) CHNC-700-2-25, and (**g**) PSPH-25. (**h**) The dark-field SEM mapping images of CHNC-700-4-25 with C, N, O, and Cu elements.

In order to investigate the morphological structure of the prepared carbon materials, the surface morphological structure of several representative CHNC and hydrochar samples was observed by SEM, revealing the influence of different reaction conditions on the surface texture of carbon materials (Figure 2). Under 700 °C (Figure 2a), CHNC-700-4-0 showed a highly porous structure with a variety of pore shapes and a rough surface. In the same reaction conditions, a more developed pore structure of CHNC-700-4-25 was observed, and its pore structure was more evenly distributed on the “Coral-like” porous carbon compared to other samples (Figure 2b). The developed pore structure and the addition of heavy-metal elements can adjust the electrochemical activity and specific surface area of the electrode material, optimizing the electrochemical performance [31,32]. With the increase in Cu content in the sample, there were obvious granular sediments in some pores of CHNC-700-4-50, which occupied the pore structure of CHNC (Figure 2c). The clogged pore can lead to a decrease in the specific surface area and active site, the blockage of ion transport, and a decrease in electrochemical stability. Figure 2d,e show the microstructures of CHNC-600-4-25 and CHNC-800-4-25, showing poor pore structure and a collapsed and blocked pore structure, respectively, indicating that both too-high and too-low activation temperatures were not conducive to the development of pore structure. For CHNC-700-2-25, the formation of less pore structure was attributed to the lower KOH ratio, which makes it difficult to effectively form a developed pore structure (Figure 2f). Figure 2g shows that the hydrochar sample had a smooth surface and relatively regular block structure, which could provide better opportunities for subsequent activation. The SEM mapping of CHNC-800-4-25, shown in Figure 2h, indicates that the C, N, O, and Cu were uniformly distributed on the surface of the carbon material, helping to maintain the structural stability of the electrochemical material and ensuring the efficiency and stability of the electrochemical reaction [33].

**Figure 3:**
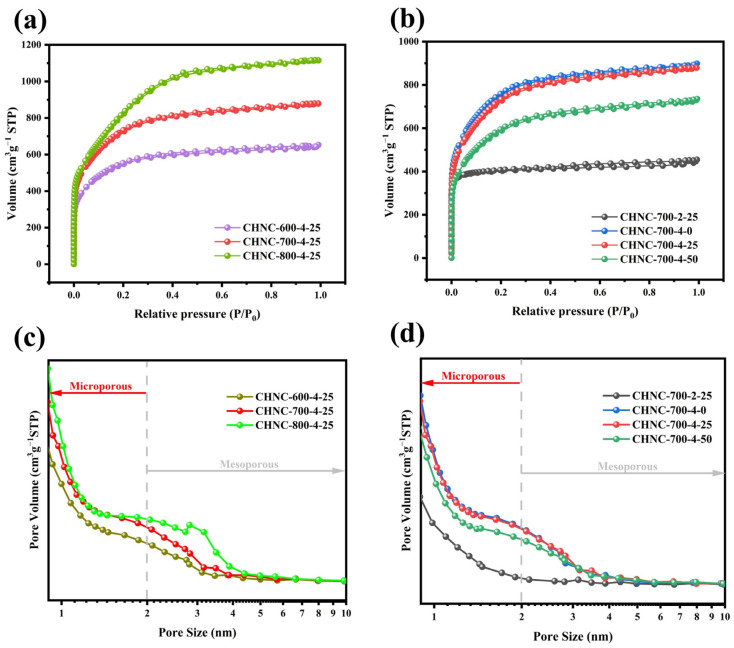
(**a**) N_2_ adsorption–desorption isotherms of CHNC-600-4-25, CHNC-700-4-25, and CHNC-800-4-25; (**b**) N_2_ adsorption–desorption isotherms of CHNC-700-2-25, CHNC-700-4-0, and CHNC-700-4-25, and CHNC-700-4-50; (**c**) pore size distribution of CHNC-600-4-25, CHNC-700-4-25, and CHNC-800-4-25; (**d**) pore size distribution of CHNC-700-2-25, CHNC-700-4-0, and CHNC-700-4-25, and CHNC-700-4-50.

The specific surface area and pore structure distribution of CHNC were analyzed by the N_2_ absorption–desorption method (Figure 3 and Table 3). All samples showed a combination of types I and IV isotherms (Figure 3a,b), and the hysteresis loops were relatively insignificant, possibly because the pore size distribution in the material was relatively uniform and microporous [34,35]. At relative low pressures (P/P_0_ < 0.05), the isotherms exhibited a sharp increase in adsorption capacity, indicating the presence of abundant micropores. However, at high relative pressures (P/P_0_ > 0.4), the adsorption and desorption processes were not completely reversible, and there was a weak hysteresis effect, which was observed as a H4 hysteresis loop with a certain mesoporous structure. All the samples had abundant hierarchical microporous (under 2 nm) and mesoporous (2–50 nm) structures (Figure 3c,d). The high specific microporous surface area increased the contact area between the electrode and the electrolyte, improving the charge transport and the electrochemical reaction rate. At the same time, the mesopore provided enough space for ion transport in the material, which means the CHNC can be used as an excellent ion transport channel with improved electrochemical performance [36,37]. The increasing specific surface areas of CHNC-600-4-25, CHNC-700-4-25, and CHNC-800-4-25 (1987.52, 2650.69, and 3019.95 m^2^ g^−1^) originated from that with the increase in temperature, the reaction of KOH becomes more and more intense, and more H_2_, CO_2_, etc., were generated, and the number of micropores and mesopores increased accordingly. Nevertheless, at high temperatures, the carbon skeleton of CHNC-800-4-25 was partly destroyed by KOH erosion, and the pore structure collapsed to form a blockage, which is negative for the electrochemical performance. For CHNC-700-2-25 and CHNC-700-4-25, the increase in the proportion of activators enabled more KOH to participate in the pyrolysis reaction and produce more H_2_, CO_2_, etc., which contributes to the generation of micropores and mesopores. In addition, it was found that with an increase in the Cu content in the sample, the specific surfaces of CHNC-700-4-0, CHNC-700-4-25, and CHNC-700-4-50 continued to decline (2746.78, 2650.69, and 2147.67 m^2^ g^−1^, respectively), which is because when the dose of copper was small, the uniform distribution of the copper particles had little influence on the pore channels. The catalytic action of the copper particles might promote the graphitization of carbon materials and, at the same time, produce gases, leaving micropores in the carbon materials. As the dose of copper increased, more copper particles blocked the pore structure on the surface of the carbon material, resulting in a decrease in the specific surface area [38,39]. Overall, CHNC had a high specific surface area and abundant pore structure. The temperature, activation ratio, and heavy-metal content could affect the structural properties of nanoporous carbon and then affect its electrochemical performance. The performance of the CHNC electrode material was well regulated by adjusting three reaction conditions.

**Figure 4:**
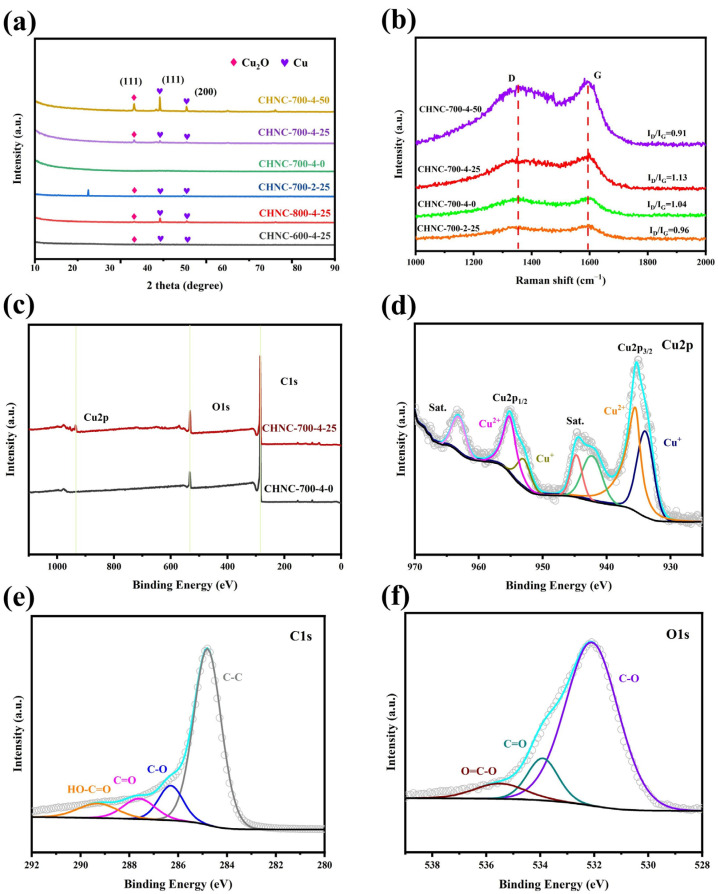
(**a**) XRD pattern of all samples, (**b**) Raman spectra of CHNC-700-2-25, CHNC-700-4-0, CHNC-700-4-25, and CHNC-700-4-50; (**c**) full-scan XPS spectra of CHNC-700-4-0 and CHNC-700-4-25, (**d**–**f**) are high-resolution XPS spectra of Cu 1s, C 1s, and O 1s for CHNC-700-4-25, respectively.

The crystallinity and phase purity of the prepared electrode material were analyzed by powder X-ray diffraction (XRD). The peaks in Figure 4a show the XRD patterns of all samples, in which the peaks of Cu_2_O (PDF#77-0199) and Cu (PDF#70-3039) were associated with the cubic phase structure. By comparing the XRD pattern of the CHNC-700-4-25 with the above standard PDF card, the peaks found at 2θ values of 43.3° and 50.5° were attributed to the (111) and (200) planes, respectively, which proved the formation of Cu. Similarly, the peak at 2θ value 36.5° was attributed to the (111) plane, demonstrating the formation of Cu_2_O. Meanwhile, for CHNC-700-4-0, no heavy metals were detected, which is in agreement with the results in Table 1. The wide peaks in the 2θ range of 15–25° were correlated with the nanoporous structure of the material.

Figure 4b shows the Raman spectra of the samples. The peaks around 1348 and 1591 cm^−1^ correspond to the D and G bands, respectively. The D band corresponds to the disordered carbon structure, while the G band is related to the graphitic carbon of the as-prepared sample [39]. The intensity ratio of the D to G peaks (I_D_/I_G_) of the CHNC-700-4-25 and CHNC-700-2-25 are 1.13 and 0.96, indicating that the high activator ratio contributed to an increase in disordered carbon [40]. The I_D_/I_G_ ratios of CHNC-700-4-0, CHNC-700-4-25, and CHNC-700-4-50 were 1.04, 1.13, and 0.91, respectively, showing that a small amount of Cu could increase the graphitization degree of the samples, while a high amount of heavy metals could reduce the disorder degree of the carbon material, thus reducing the specific surface area and electrochemical performance of the electrode material.

The chemical and electronic states and the elemental compositions of CHNC-700-4-0 and CHNC-700-4-25 were analyzed by X-ray photoelectron spectroscopy (XPS). The full-scan XPS spectra in Figure 4c revealed that CHNC-700-4-25 contains Cu (Cu 2p), O (O 1s), and C (1s). Figure 4d shows the high-resolution Cu 2p spectrum of CHNC-700-4-25 by deconvoluting the high-resolution spectra by the Gaussian fitting method. The two peaks at binding energies of 933.97 and 952.97 eV were ascribed to Cu^+^ in Cu_2_O [40]. Similarly, the other two peaks at binding energies of 935.51 and 955.17 eV were ascribed to Cu^2+^ in CuO. Moreover, the two noticeable satellite peaks at 942.20 and 944.71 eV were related to CuO, and the peaks at 963.12 eV were related to Cu_2_O [41]. Figure 4e shows the O1s signal of CHNC-700-4-25, which were deconvoluted into three oxygen-based components: C-O (532.2 eV), C=O (533.8 eV), and -COOR (535.7 eV). The oxygen-containing groups could increase the force between CHNC and aqueous electrolytes and improve the wettability and interfacial stability of the carbon materials [42,43]. Figure 4f shows the existence of deoxygenated carbon (C-C) at 284.80 eV, oxygenated carbon (C-O, 286.2 eV); C=O, 287.6 eV; HO-C=O, 289.2 eV). The XPS analysis of CHNC-700-4-25 further supports the XRD results and confirms the successful synthesis of CHNC as a copper-hybrid material.

### 2.3. Electrochemical Performance Evaluation

#### 2.3.1. Electrode Test

**Figure 5:**
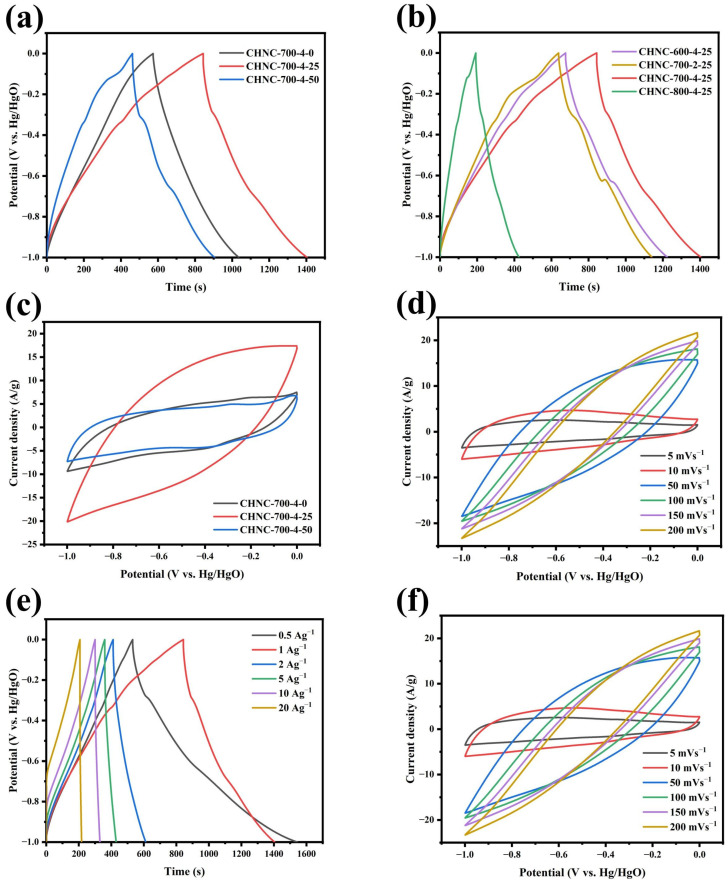
Electrochemical performance of samples in the three-electrode system: (**a**,**b**) GCD curves for all samples, (**c**,**d**) CV curves for all samples, (**e**) GCD curves of CHNC-700-4-25 at different densities from 0.5 to 20 A g^−1^, and (**f**) CV curves of CHNC-700-4-25 at different scanning rates from 5 to 200 mV s^−1^.

To explore the charge storage behavior of the CHNC in supercapacitors, cyclic voltammetry (CV), galvanostatic charge/discharge (GCD) tests, and electrochemical impedance spectroscopy (EIS) were first performed on a three-electrode system and two-electrode system in a 6M KOH electrolyte solution. The GCD curves of CHNC at a current density of 1 A g^−1^ are presented in Figure 5a,b. All GCD curves exhibited an almost isosceles triangular shape, indicating excellent charge/discharge characteristics and electrochemical reversibility. For the samples containing heavy metals, we found that the GCD curve would bend slightly during the charge and discharge process, especially on the samples of CHNC-700-2-25 and CHNC-700-4-50, while this was not the case on CHNC-700-4-0. This is because the presence of Cu and Cu_2_O changes the microstructure of electrode materials, such as the specific surface area and pore structure, and participates in the electrochemical reaction as an active site, thus affecting the efficiency and path of the electrochemical reaction, resulting in the bending of the GCD curve [44]. At the scan rate of 5 mV s^−1^, different samples showed different CV curve shapes. CHNC-700-4-25 exhibited the best asymmetric rectangular shapes (Figure 5c,d), indicating a fast electrochemical reaction and the behavior of dominant EDLCs. For the CHNC-800-4-25, CHNC-700-2-25, and CHNC-700-4-50, the high temperature, lower activation ratio, and high heavy-metal content caused structural changes in the carbon material. As a result, the structural parameters such as grain size, specific surface area, and pore size of the electrode material change, resulting in the distortion of the CV curve. Notably, CHNC-700-4-25 exhibited the largest CV integral area and the longest charge/discharge time, confirming the highest charge storage capacity. Figure 5e shows the GCD curves of CHNC-700-4-25 at different current densities. All GCD curves maintained relative symmetry, revealing great electrochemical reversibility, the stability of electrode materials, and the effective transfer of ions at the electrode-electrolyte interface. The CV curves of CHNC-700-4-25 at scan rates (5–200 mV s^−1^) are shown in Figure 5d. The CV curve approximately maintained a rectangular shape at low scan rates (5–10 mV s^−1^), indicating its quick ion transport capability. With the increase in scanning rate, the CV curve shows a tendency to distort because, at a high scanning rate, ion diffusion is limited, resulting in uneven electrode surface charge and CV curve deformation [45,46].

**Figure 6:**
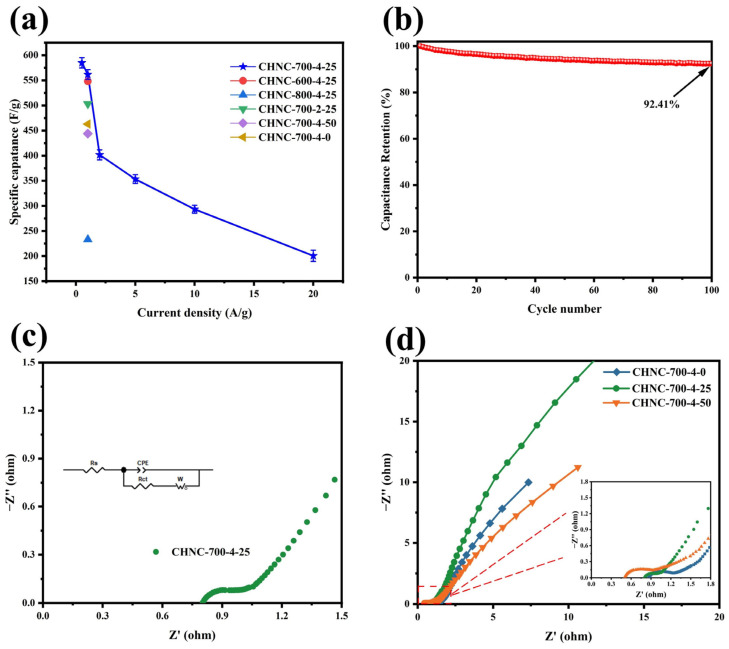
(**a**) The specific capacitance of all samples at different current densities: (**b**) The electrochemical cycle test of CHNC-700-4-25 was tested at 10 A g^−1^, (**c**) the equivalent circuit diagram of CHNC-700-4-25, and (**d**) the Nyquist plots of three samples of different heavy-metal contents.

The specific capacitance at different current densities (0.5–20 A g^−1^) was calculated according to the discharge curve and Equation (1). Figure 6a summarizes the specific capacitance of different CHNC materials at different current densities. Apparently, CHNC-700-4-25 shows the ultrahigh specific capacitance (562 F g^−1^) at a current density of 1 A g^−1^, indicating its phenomenal charge storage capacity. The cyclic stability of CHNC-700-4-25 was tested at 10 A g^−1^ (Figure 6b). At the beginning of the cycle, it shows a decaying trend and can still maintain 92.41% capacity after 10,000 cycles, demonstrating its excellent cyclic performance. The blockage and collapse of pore structures, changes in surface oxygen-containing functional groups, loss of active sites, and deposition of impurities on electrode surfaces can all cause a decline in capacitance performance [47]. The EIS measurement of CHNC was studied to evaluate the electrochemical performance. The equivalent circuit diagram is shown in Figure 6c, where R_s_ denotes the equivalent series, and R_ct_ represents the charge-transfer resistances [48]. Notably, within the low-frequency spectrum, a trendline with a 45° inclination was observed, which corresponds to the Warburg impedance (W). The Warburg impedance is intimately linked to the diffusion coefficient of the charge-carrying substance within the solution [49]. The Nyquist plots shown in Figure 6d show approximately vertical lines in the low-frequency region, revealing their great EDLC behavior [50]. The values of the R_s_ and R_ct_ for various samples are compiled in Table S1 (Supporting Information). Specifically, CHNC-700-4-25 exhibited significantly reduced R_s_ (0.805 U) and R_ct_ (0.081 U), which is indicative of its low internal resistance and superior conductivity. This diminished internal resistance further minimized thermal losses, potentially extending the operational lifespan of CHNC-700-4-25.

#### 2.3.2. Test on Symmetric Supercapacitor CHNC-700-4-25//CHNC-700-4-25

**Figure 7:**
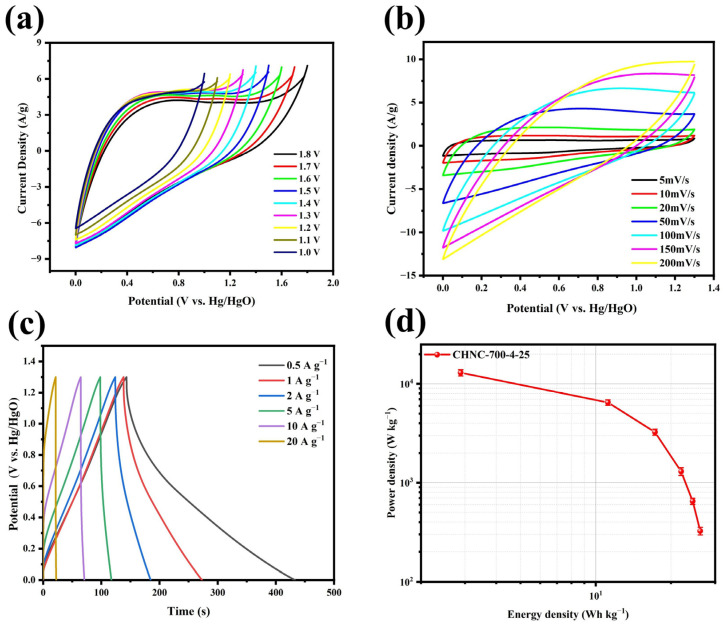
(**a**) CV curves of CHNC-700-4-25 at different open-circuit voltages, (**b**) CV curves of CHNC-700-4-25 at 1.3 V at open-circuit voltage, (**c**) GCD curves of CHNC-700-4-25 at different current densities, and (**d**) the relationship of CHNC-700-4-25 between energy density and power density.

To further evaluate the performance of CHNC-700-4-25, the symmetric supercapacitor CHNC-700-4-25//CHNC-700-4-25 was assembled using 6 M KOH as the electrolyte. The CV test was performed in the voltage window of 0–1.8 V at a scan rate of 50 mV s^−1^ to test the rate performance of the symmetric supercapacitor (Figure 7a). The emergence of the anode current in the CV curve upon increasing the voltage window to 1.3 V suggested the stable operation of the symmetric supercapacitor within the voltage range of 0–1.3 V. As depicted in Figure 7b, the CV curves exhibited a well-maintained rectangular shape across varying scan rates, thereby validating the superior capacitive properties of CHNC materials [46]. Additionally, the GCD curves (Figure 7c) revealed an isosceles triangular profile at different current densities, indicative of the symmetric supercapacitor’s excellent rate capability and rapid reversibility during charging and discharging. Notably, the specific capacitance of the symmetric supercapacitor attained a peak value of 416 F g^−1^ at a current density of 1 A g^−1^ while still maintaining a commendable capacity of over 100 F g^−1^ even at a high current density of 10 A g^−1^ (Figure S1, Supporting Information). To assess the comprehensive performance of the CHNC-700-4-25//CHNC-700-4-25 configuration, the relationship between the energy density and power density was depicted in the GCD curves through Equations (3) and (4), respectively (Figure 7d). It is worth noting that the energy density achieved a peak value of 26.15 W h kg^−1^ at a power density of 324.87 W kg^−1^, and it maintained a commendable value of 11.18 W h kg^−1^, even at a significantly higher power density of 6490.42 W kg^−1^, demonstrating the exceptional electrochemical characteristics and promising applications of the investigated configuration. As can be seen in Table 4, the energy storage capacities of CHNC-700-4-25 were superior to many other reported biomass-based carbons.

## 3. Materials and Methods

### 3.1. Materials and Reagents

Waste pine sawdust was collected from a wood processing factory in Beijing (China). CuSO_4_·5H_2_O, acetylene black, and polytetrafluoroethylene (PTFE) were purchased from Aladdin Industrial Corporation (Shanghai, China). The other chemical reagents and materials used in this study included nickel foam used to prepare the working electrodes, KOH (AR, Tianjin Kemio Chemical Reagent Co., Ltd., Tianjin, China) as the activating agent; hydrochloric acid (HCl, 38%), procured from Sinopharm Chemical Reagent Co., Ltd., Shanghai, China; and anhydrous ethanol from Anhui Ante Food Co., Ltd. (AR) (Suzhou, China). And the equipment used for carbon preparation in this study included a tubular furnace (CHY-1200, Henan Chengyi Equipment Technology Co., Ltd., Zhengzhou, China) and a press machine (YLJ-5T, Hefei Kejing Material Technology Co., Ltd., Hefei, China).

### 3.2. Synthesis of Copper-Ion Hybrid Nanoporous Biochar

**Figure 8:**
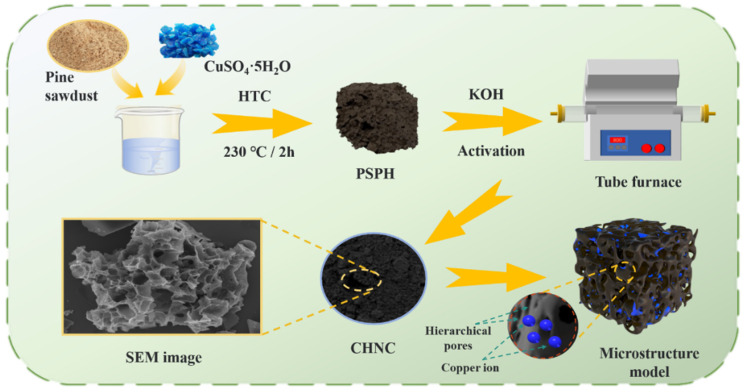
Schematic diagram of the preparation process of Cu-ion hybrid nanoporous carbon (CHNC).

Figure 8 details the synthesis of CHNC. Firstly, the waste pine sawdust was dried in an oven at 105 °C to a constant weight for 5 h. The dried pine sawdust was then ground further in the crusher and passed through an 80-mesh sieve. To accurately adjust and analyze the effect of heavy metals throughout the experiment, the Cu-contaminated plants used in the reactions were synthesized using appropriate amounts of Cu and crushed pine sawdust according to the reported methods [22,26,27]. The desired amounts of crushed pine sawdust and CuSO_4_·5H_2_O solutions were mixed in a plastic bottle and shaken vigorously at 150 rpm for 6 h. The resulting mixtures (0, 25, 50 mg Cu/g biomass) were used for the HTC reaction. A total of 150 mL of ultrapure water and the desired amounts of Cu-contaminated plants (composed of 15 g of biomass and the desired amount of Cu) were loaded into a 250 mL autoclave and then heated up to 230 °C for 120 min. After the reactor cooled down to room temperature, the HTC products were filtered with deionized water three to four times and poured into a Petri dish, which was then dried to a constant weight at 80 °C for 12 h. The dried solid HTC products were named pine sawdust pre-carbonized hydrochar (PSPH-M), where M represented the Cu content (mg) per gram of biomass (M = 0, 25, or 50). Using KOH as the activator, pine sawdust pre-carbonized hydrochar was mixed with KOH at weight ratios of 1:X (X = 2, 3, or 4) in a mortar and ground into powder. The mixture was then placed in a nickel crucible and subjected to pyrolytic activation in a nitrogen atmosphere (25 mL min^−1^). The temperature was increased at a heating rate of 2 °C min^−1^ to 700 °C and held for 1 h. Then, the sample was naturally cooled to room temperature in the tubular furnace under a nitrogen atmosphere. The activated sample was filtered with 0.1 M hydrochloric acid three to four times and washed with deionized water until neutralized. The washed sample was dried in a Petri dish at 80 °C to a constant weight. The activated samples were named CHNC-T-X-Y, where T represented the activation temperature (T = 600 °C, 700 °C, or 800 °C), X represented the weight ratios of KOH and PSPH-M (X = 2, 3, or 4), and Y represented the Cu content (mg) per gram of biomass (M = 0, 25, or 50). Under the same reaction conditions, all experiments were conducted three times to ensure reproducibility.

### 3.3. Materials Characterization

The morphological structure of the samples was examined utilizing a field emission scanning electron microscope (SEM, Gemini SEM 500, Carl Zeiss Optics Co., Ltd., Jena, Germany), and energy diffraction spectroscopy (EDS) was used to investigate the elemental composition. The specific surface area of the nanoporous biochar was analyzed employing the multipoint Brunauer–Emmett–Teller (BET, V-Sorb 2800P, Guoyi Precision Measurement Technology Co., Ltd., Beijing, China) method. The total pore volume (V_t_) was calculated based on the nitrogen adsorption measurements at a relative pressure of 0.99. The pore size distribution was determined using the Barrett–Joyner–Halenda method. The elemental contents of carbon (C), hydrogen (H), and nitrogen (N) in the samples were analyzed with an elemental analyzer (Vario EL cube, Elementar Analysen systeme GmbH., Frankfurt, Germany). The surface elemental composition and chemical groups of the nanoporous biochar samples were exhaustively characterized via X-ray photoelectron spectroscopy (XPS, AXIS SUPRA^+^, Kratos Analytical Ltd., Manchester, UK). The crystal structure was systematically investigated using an X-ray diffractometer (D8-ADVANCE, Brook Technology Co., Ltd., Berlin, Germany). Furthermore, Raman spectroscopic analysis was performed with a laser micro-Raman spectrometer (Renishaw, Renishaw plc., Gloucestershire, UK).

### 3.4. Electrochemical Measurements

#### 3.4.1. Electrode Preparation

For the three-electrode configuration, CHNC-T-X-Y, acetylene black, and polytetrafluoroethylene (PTFE) binder were mixed in a specific mass ratio of 8:1:1. The mixture was dispersed in anhydrous ethanol, serving as an emulsion-breaking agent, and thoroughly ground using a mortar and pestle to ensure homogeneity. The resulting composite, once uniformly ground, was then coated onto a nickel foam sheet (measuring 1.0 × 1.0 cm^2^) and subsequently pressed into a thin sheet under moderate pressure (10–20 MPa). For the two-electrode system, with the same ratio of CHNC-T-X-Y, acetylene black, and PTFE, a polypropylene diaphragm was chosen for the construction of the symmetrical capacitors.

#### 3.4.2. Electrochemical Performance Test

The electrochemical evaluations encompassed cyclic voltammetry (CV), galvanostatic charge/discharge (GCD), and electrochemical impedance spectroscopy (EIS) reactions and cycling stability tests at room temperature executed on an electrochemical workstation (CHI760E Chenhua, Shanghai, China). In the three-electrode setup, Pt and HgO/Hg served as the counter and reference electrodes, respectively, with 6 M KOH as the electrolyte. For the two-electrode configuration, a simulated double-layer capacitor was assembled with two identical 1.0 × 1.0 cm^2^ carbon electrodes separated by a polypropylene diaphragm, also utilizing 6 M KOH. EIS measurements were conducted at frequencies spanning from 0.1 Hz to 100 kHz, with an amplitude of 5 mV.

From the galvanostatic charge/discharge curve, the specific capacitance *C_sp_* (F g^−1^) of the three-electrode system can be calculated as follows:

(1) $$C_{\mathrm{sp}}\;=\frac{I\;\times \;∆t}{m\;\times \;∆V}$$

where *I* (A) is the discharge current, ∆*t* (s) refers to the discharge time, *m* (g) is the mass of active material on the working electrode, and ∆*V* (V) refers to the potential change range during the discharge.

The formula for the specific capacitance of the two-electrode system can be calculated by Equation (2).

(2) $$C_{\mathrm{sp}}\;=\frac{4\;\times \;I\;\times \;∆t}{m\;\times \;∆V}$$

where *C_sp_* (F g^−1^) is the specific capacitance of a single electrode.

The calculation of energy density *E* (W h kg^−1^) and power density *P* (W kg^−1^) can be realized on the basis of Equations (3) and (4), respectively.

(3) $$E=\frac{C_{\mathrm{sp}}\;\times {(∆V)}^{2}}{8\;\times \;3.6}$$

(4) $$P=\frac{\;E\;\times \;3600}{∆t}$$

## 4. Conclusions

In summary, the present work demonstrated a facile and value-added utilization of Cu-contaminated biomass by HTC-coupling pyrolytic activation. The specific capacitance of CHNC-700-4-25 reached 562 F g^−1^ at 1 A g^−1^, and after 10,000 cycles, its retention rate was higher at 92.41% at a current density of 10 A g^−1^. Notably, the symmetrical supercapacitor CHNC-700-4-25//CHNC-700-4-25 exhibited an energy density of 26.15 W h kg^−1^ when the power density was 324.87 W kg^−1^ in 6 M KOH electrolyte, which indicated an exceptional overall supercapacitor performance. Interestingly, we found that the optimal concentration of copper ion significantly increased the specific capacitances from 463 to 562 F g^−1^ of CHNC, indicating that the ultrahigh-energy storage performance of CHNC is tunable by the adjustment of Cu content during HTC and pyrolysis. Heavy metals improved the energy density, cycle life, and charge/discharge performance of batteries by optimizing the material structure and performance, promoting the development of electrochemical energy storage technology, and providing ideas and foundations for the development of new energy storage devices. At the same time, it provided an environmentally friendly and sustainable approach for the resource utilization of biomass contaminated with heavy metals.

## Figures and Tables

**Table 1:** Cu content and distribution in various samples.

Sample	Content of Cu (mg/g)	Yield ^a^ (%)	Original Cu Distribution (%)
PSPH-0	0	59.96	0
PSPH-25	49.08	49.56	97.31
PSPH-50	91.63	47.75	87.48
CHNC-600-4-25	10.62	17.65	7.51
CHNC-700-2-25	17.10	20.44	14.53
CHNC-700-4-0	0	9.33	0
CHNC-700-4-25	26.47	12.85	13.64
CHNC-700-4-50	49.79	14.08	14.32
CHNC-800-4-25	68.58	6.78	18.59

^a^ Based on dried raw biomass.

**Table 2:** Elemental analysis results and yield of different samples.

Sample	N (%)	C (%)	H (%)	O (%) ^a^	H/C	O/C
PSPH-0	2.06	58.56	5.96	33.42	1.22	0.43
PSPH-25	1.96	62.22	5.05	30.77	0.97	0.37
PSPH-50	1.56	61.99	5.27	31.18	1.02	0.38
CHNC-600-4-25	1.76	62.03	3.27	32.94	0.63	0.40
CHNC-700-2-25	2.35	66.23	3.366	28.054	0.61	0.32
CHNC-700-4-0	1.7	87.62	1.464	9.216	0.20	0.08
CHNC-700-4-25	1.72	81.67	1.301	15.309	0.19	0.12
CHNC-700-4-50	2.77	63.04	2.216	31.974	0.42	0.38
CHNC-800-4-25	2.53	87.4	2.238	7.832	0.31	0.07

^a^ Calculated by difference.

**Table 3:** Porosity parameters and specific capacitances of the CHNCs at 1 A g^−1^.

Sample	S_BET_^a^ (m^2^ g^−1^)	V_total_^b^ (cm^3^ g^−1^)	V_micro_^c^ (cm^3^ g^−1^)	V_micro_/V_total_ (%)	V_meso_ ^d^ (cm^3^ g^−1^)	Capacity (F g^−1^)
CHNC-600-4-25	1987.52	1.12	0.83	0.74	0.29	548
CHNC-700-2-25	1620.26	0.73	0.62	0.85	0.11	503
CHNC-700-4-0	2746.78	1.54	1.13	0.73	0.41	463
CHNC-700-4-25	2650.69	1.52	1.09	0.72	0.43	562
CHNC-700-4-50	2147.67	1.29	0.89	0.69	0.4	444
CHNC-800-4-25	3019.95	2.12	1.22	0.58	0.9	233

^a^ S_BET_ is a specific area by the BET method at P/P0 = 0.001−0.05. ^b^ V_total_ is the total pore volume at P/P0 = 0.99. ^c^ V_micro_ is the micropore volume and is evaluated by the t-plot method. ^d^ V_meso_ is the mesopore volume and is evaluated by the BJH method.

**Table 4:** Comparison of SSA and electrochemical properties between CHNC-700-4-25 and other biomass electrode materials.

Biomass-Derived Electrode Materials	SSA (m^2^ g^−1^)	Specific Capacitance (F g^−1^)	Current Density (A g^−1^)	Energy Density (W h kg^−1^)	Power Density (W kg^−1^)	Cycle Stability (%)	Refs.
CHNC-700-4-25	2650.69	562	1	24.39	649.98	92.41/10,000 cycles	This work
AHCT_8_C_180_ (Paper mulberry fruit juice; O and P doped)	3543.8	428	0.5	9.56	125	93.7/10,000 cycles	[51]
HAPC (Leaves of *Enhydra fluctuant*; multi-heteroatom doped)	1082.8	428	1	73.88	5.11	94/10,000 cycles	[52]
ASC-3 (Aucklandia lappa straw)	2427	341	0.5	19.59	52.03	89.47/10,000 cycles	[53]
WBPC_1-2-600_ (The wheat bran)	544	312	0.5	16.63	250	91.2/3000 cycles	[54]
CS-7 (Wooden chopsticks)	83	185	0.3	0.083	300	96/10,000 cycles	[55]
LSB-700 (litchi seeds)	372.02	190	1	24.6	600	90/10,000 cycles	[56]
Act_OFSCBC (onion flower seed)	2538.31	200	1	21.94	500	74.4/10,000 cycles	[57]
CAC_700-4_ (corncob)	1945.7	260	1	14.3	250	95.5/10,000 cycles	[58]

## Data Availability

Data are contained within the article and Supplementary Materials.

## Supplementary Materials

The following supporting information can be downloaded at: https://www.mdpi.com/article/10.3390/ijms26020569/s1.
