# Supplementary material for: Cu-Ion Hybrid Porous Carbon with Nanoarchitectonics Derived from Heavy-Metal-Contaminated Biomass as Ultrahigh-Performance Supercapacitor
Source: Int J Mol Sci. 2025 Jan 10;26(2):569. doi: 10.3390/ijms26020569 (PMC11766103; doi:10.3390/ijms26020569)
Supplement: Supplementary file 1 [file ijms-26-00569-s001.zip › ijms-3396163-supplementary.pdf]

Supporting Information

Cu-Ion Hybrid Porous Carbon with Nanoarchitectonics Derived from Heavy-Metal-Contaminated Biomass as Ultrahigh-Performance Supercapacitor

Jieni Wang[1,2], Xiaobo Han[1,2], Shuqin Zhang[1,2], Haodong Hou[1,2], Chenlin Wei[1,2], Chenxiao Liu[1,2], Leichang Cao[1,2*], Jinglai Zhang[1*], Li Wang[1], Shicheng Zhang[3]

[1] Henan Key Laboratory of Protection and Safety Energy Storage for Light Metal Materials, College of Chemistry and Molecular Sciences, Henan University, Kaifeng 475004, China

[2] Miami College, Henan University, Kaifeng 475004, China

[3] Department of Environmental Science and Engineering, Fudan University, Shanghai, 200433, China

[*] Corresponding Author: clch666@henu.edu.cn; zhangjinglai@henu.edu.cn

Number of Pages: 4
Number of Figures: 1
Number of Tables: 1

The cyclic voltammetry (CV) and galvanostatic charge/discharge (GCD) curves were measured in a potential window (-1~0 V) at different scan rates (5~200 mV s^{-1}) and current densities (0.5~20 A g^{-1}), respectively. Electrochemical impedance spectra (EIS) were recorded in an open circuit potential over a frequency range of 0.01 Hz to 100 kHz.

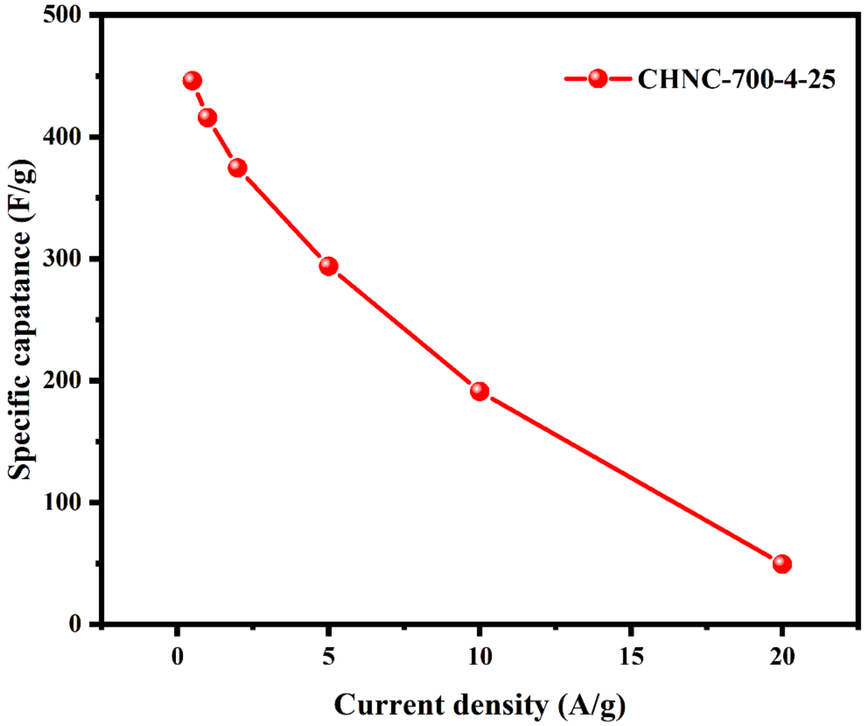

Figure S1. Specific capacitance of the symmetric supercapacitor at different current densities.

Table S1. The values of the R_s and R_{ct} for various samples.

Sample	R_s	R_{ct}
CHNC-600-4-25	1.218	0.09422
CHNC-700-2-25	1.208	0.08328
CHNC-700-4-0	0.8821	0.1179
CHNC-700-4-25	0.8050	0.0806
CHNC-700-4-50	0.5153	0.1608
CHNC-800-4-25	1.828	0.08491
